# The radiologic progression of ameloblastomas

**DOI:** 10.4102/sajr.v27i1.2668

**Published:** 2023-05-31

**Authors:** Lene Merbold, Chané Smit, Jason Ker-Fox, Andre Uys

**Affiliations:** 1 Department of Oral and Maxillofacial Pathology, Faculty of Health Sciences, University of Pretoria, Pretoria, South Africa; 2 Private Statistician, Cape Town, South Africa; 3 Department of Anatomy, Faculty of Health Sciences, University of Pretoria, Pretoria, South Africa

**Keywords:** ameloblastoma, delayed treatment, neoplasm, maxillofacial radiology, benign odontogenic neoplasm, progression

## Abstract

**Background:**

In developing countries, many diagnosed cases of ameloblastoma (AB) have a significant delay in receiving treatment because of patient factors and healthcare facility constraints.

**Objectives:**

The radiologic progression of ABs with delayed treatment was analysed using panoramic radiographs and cone-beam computed tomography imaging.

**Method:**

Histopathologically confirmed cases of AB with follow-up radiographs indicating no treatment were retrospectively reviewed over a study period of 10 years. Fifty-seven cases with 57 initial and 107 follow-up radiographs were included. Each follow-up radiograph was analysed for changes in borders, locularity, effects on surrounding structures and lesion size.

**Results:**

There was a general increase in poorly-demarcated lesions, with seven cases transforming from an initial unilocular to a multilocular appearance. At follow-up, there was an increase in cortical thinning and cortical destruction. Ameloblastomas presented with a three-fold increase in average size from the initial to follow-up visits. Regression analysis showed a statistically significant relationship between lesion duration and length (*p* = 0.001). A statistically significant relationship existed between duration and overall lesion dimensions when only the first and last observations per patient were used (*p* = 0.044).

**Conclusion:**

Considering the aggressive nature and unlimited growth potential, ABs with delayed treatment may show extensive growth, complicating their eventual management.

**Contribution:**

This study aimed to raise awareness of the importance of the timeous management of patients with AB by highlighting the detrimental effects of delayed treatment.

## Introduction

Ameloblastoma (AB) is a benign odontogenic neoplasm arising from epithelial remnants of the dental lamina.^1^ The aetiopathogenesis of ABs has not yet been fully elucidated, but mutations in the genes involved in the mitogen-activated protein kinase (*MAPK*) pathway have been implicated in 90% of ABs.^2^ Clinically ABs present as slow-growing, painless, expansile masses that can exhibit accelerated growth.^1^ Facial swelling and asymmetry may arise because of tumour enlargement over time. Intra-orally, malocclusion, ill-fitting dentures and teeth mobility may occur,^3^ with advanced cases showing restricted mouth opening, difficulty with mastication and airway obstruction.^1^ Although infrequent, pain, paraesthesia and pathologic fractures may be accompanying signs and symptoms.^4^

Because of the asymptomatic nature, patients often only seek medical care when a facial deformity is noticeable.^5^ The treatment of AB is controversial. There are two main surgical approaches, namely conservative and radical. The former involves enucleation, curettage or cryosurgery of the bony cavity,^5^ while radical surgery includes surgical resection with 1 cm – 2 cm clear bony margins. The margin marked for resection is defined as the distance from the radiologic margin predicted to be disease free.^6^ This is recommended because of the bony infiltration of neoplastic cells beyond the radiologic margins.^6,7^ Patients who receive conservative treatment have a higher propensity for recurrence (90%) than those who receive radical treatment (5%).^8,9^ Post-operative follow-up for all AB cases is critical as over 50% of recurrences can occur 5 years post-treatment, with some presenting as early as 2 years after surgical intervention.^5^ Because of high recurrence rates and the possibility of malignant transformation,^8^ the current treatment of choice is radical wide surgical excision.^8,10,11^ Exceptions to this treatment approach are the luminal and intraluminal variants of unicystic ABs, which may be managed with conservative treatment.^6^ Radical surgery is frequently performed in a single-stage procedure to restore normal function, aesthetics and to decrease hospital inpatient stay.^12^

In developing countries, patients with ABs often present with lesions reaching a considerable size before seeking care.^13^ Considering the unlimited growth potential, delayed treatment of ABs may show extensive and progressive growth, complicating their management.^9^ Ultimately, if ABs are delayed in treatment, they can continue to enlarge, leading to encroachment of anatomical structures, decreased function, closure of the airway, metabolic abnormalities and can, in rare instances, be fatal.^6^

The aim of the study was to analyse the radiological progression of ABs, analysed on follow-up panoramic radiographs (PRs) and cone-beam computed tomography (CBCT) imaging. This study aimed to raise awareness of the importance of the timeous management of patients with AB by highlighting the detrimental effects of delayed treatment.

## Research methods and design

All histopathologically confirmed cases of AB with follow-up radiographs consisting of PRs and/or CBCT images were retrospectively reviewed over a 10-year period (2012 to 2021). Accordingly, diagnosed cases from the Department of Oral and Maxillofacial Pathology were searched on the radiographic database within the Section of Diagnostic Imaging. For the purpose of this study, ABs with delayed treatment included cases with follow-up radiographs showing no radiologic signs of treatment for the tumour. Teeth lost because of extraction, mobility or tumour expansion was not considered as treatment.

During the 10-year study period, a total of 781 cases of AB were histologically diagnosed within the Department of Oral and Maxillofacial Pathology. External referral cases were excluded from the current study because the radiographs were not on the hospital’s digital radiographic database. In other instances, no follow-up radiograph could be detected, and the patient was therefore deemed lost to follow-up. Cases with significant positioning errors on the PRs were excluded. Lastly, cases that only had an initial and post-operative radiograph were excluded. A total of 724 cases were excluded, with 57 cases included in the final sample. The demographic information of included cases was collected from the patient’s hospital records.

The radiologic features were analysed by two clinicians with experience in the field of maxillofacial radiology, with any disagreements resolved by consensus. Panoramic radiograph examinations were analysed with Cliniview^©^ software for radiographs performed on the Instrumentarium Dental unit (Orthopantomograph^®^/Orthoceph^®^ OP200D/OC200D, Finland) and Sidexis^©^ software for radiographs performed using Sirona Dental Systems, Orthophos XG, Germany. These radiographs were performed using the manufacturer’s instructions for recommended exposure for adults and children. All PR measurements were corrected for magnification. All CBCT images were performed on Planmeca ProMax 3D (Helsinki, Finland) and were evaluated by the principal investigator using Romexis software (Romexis version 6.0.1.812). The exposure setting for each CBCT scan differed based on the field of view and patient parameters. Cone-beam computed tomography examinations do not exhibit magnification because of the isotropic reconstruction of the volumetric data. All images were viewed in a dimly lit room on a radiology reporting monitor (Barco Diagnostic monitor with two-megapixel resolution). Each image was optimised for assessment by adjusting the sharpness, density and contrast.

A standardised patient positioning and procedure is maintained for all PRs at the institution. Panoramic radiographs with significant positioning errors and dimensional aberrations were excluded from the evaluations. For the CBCT measurements, the patient’s orientation could be standardised after acquisition. The medio-lateral head tilt was orientated in the coronal slice of the scan by aligning an imaginary line running from the crista galli to the midsagittal suture of the maxilla, parallel to the sagittal orientation line. The superior-inferior head tilt was orientated in the midsagittal slice by placing the imaginary line from the anterior to the posterior nasal spine parallel to the axial orientation line. A PR was reconstructed from the volumetric data based on this positioning. This was done by manually drawing the focal trough running from the condylar head on the right, following the central points corresponding to the curve of the mandible, to the contralateral condylar head. The focal trough’s widest setting was used, equating to 19.9 mm.

**FIGURE 1:**
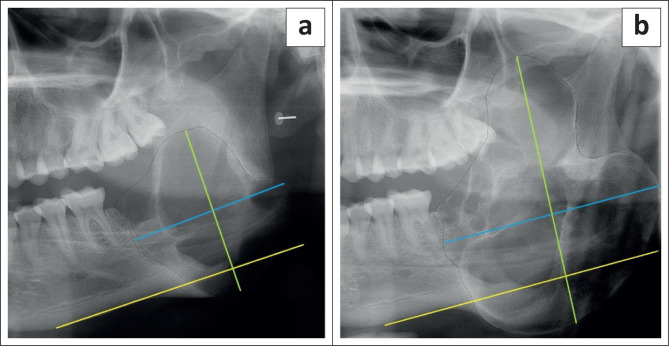
Size measurement of an ameloblastoma on panoramic radiograph at initial presentation (a) and after 17 months (b). The yellow line represents the inferior border of the mandible. The blue line represents the anterior-posterior dimension and the green line the superior-inferior dimension.

**FIGURE 2:**
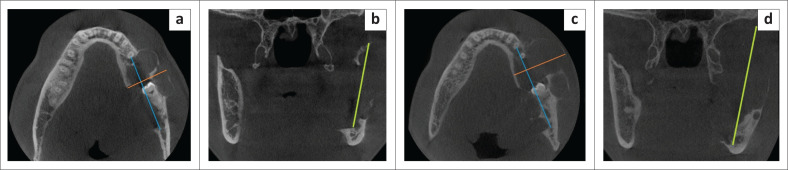
Size measurement of an ameloblastoma on cone-beam computed tomography at initial presentation (a, b) and after 11 months (c, d). The blue line represents the anterior-posterior dimension, the green line the superior-inferior dimension, and the orange line the medial-lateral dimension.

For the radiologic analysis, lesions were classified as anterior (canine to canine region) or posterior (distal to the first premolar). The borders, locularity, density and effects on surrounding structures were analysed for each initial and all follow-up radiographs. Lastly, the size was calculated by measuring the anterior-posterior, superior-inferior and medial-lateral dimensions. The anterior-posterior dimension was defined as the longest dimension of the lesion measured by a line running parallel to the inferior border of the mandible in millimetres (mm) on PR. On CBCT imaging, this was measured at the greatest dimension on the axial slice. In cases where the lesion followed a curvilinear pattern in the mandible when both the anterior and posterior regions were involved, this measurement was done on the reconstructed PR. The superior-inferior dimension was defined as the highest dimension of the lesion measured by a line perpendicular to the inferior border of the mandible in millimetres (Figure 1). On CBCT imaging, this was measured at the widest dimension on the coronal view. The medial-lateral dimension was only assessed on CBCT images and was defined as the widest dimension of the lesion measured by a line running perpendicular to the length as measured on the axial view (Figure 2). Calibration for the magnification of PRs was performed for both software systems to have measurements comparable to the CBCT unit.

The data analysis was done in the form of descriptive analysis and confirmatory data analysis. A univariate frequency table was constructed for each categorical variable, showing the percentage breakdown and distribution of the cases according to the variable parameters. Additionally, multivariate tables were constructed to highlight the interaction of categorical variables prior to determining the statistical significance thereof. The association between time (continuous variable) and the various radiologic features of ABs (categorical variable[s]) was evaluated using the Wilcoxon Rank-Sum test, along with the two-sample *t*-test being used as a reasonability check. Correlations with a two-sided asymptotic significance (*p*-value) of less than 0.05 were deemed statistically significant. A linear regression analysis was done to determine the change in lesion dimensions over time. These tests aimed to assess whether there was any relationship between change in time and growth measured using the radiologic dimensions.

The inter- and intra-examiner reliability for measurements was evaluated using the interclass correlation coefficient (ICC) as the data consisted of continuous variables. To assess the reliability of evaluations, the principal investigator re-evaluated 25 cases with their follow-up radiographs after 1 month to assess the intra-examiner reliability. Additionally, 15 randomly selected cases with their follow-up radiographs were evaluated by a second calibrated investigator in order to determine the inter-examiner reliability.

### Ethical considerations

The study was conducted following approval by the Faculty of Health Sciences Research Ethics Committee, University of Pretoria (571 of 2021). All procedures followed were in accordance with the ethical standards of the responsible committee on human experimentation (institutional and national) and with the Helsinki Declaration of 1975, as revised in 2008.

## Results

Table 1 summarises the main demographic features. The mean age of patients at initial presentation was 34 years (range: 7–79), with a peak incidence in the third and fourth decades of life. The sample included 26 males and 31 females showing a slight female predilection of 1:1.2.

**TABLE 1:** Demographic features and location of the 57 untreated cases of ameloblastoma diagnosed over the 10-year study period.

Age and location	Results (*n* = 57			
	Mean	Range	*n*	%
**Age (years)**	34	7–79	-	-
Male	33	7–62	-	-
Female	35	11–79	-	-
**Location maxilla**	-	-	3	5.26
Anterior maxilla	-	-	1	1.75
Posterior maxilla	-	-	1	1.75
Anterior and posterior maxilla	-	-	1	1.75
**Mandible**			54	94.73
Anterior mandible	-	-	3	5.26
Posterior mandible	-	-	30	52.63
Anterior and posterior mandible	-	-	21	36.84

The location of included cases of AB showed that maxillary lesions (5.3%) were a rare finding. Ameloblastomas had a mandibular predilection (94.7%), with 89.5% of all cases involving the posterior mandible.

All available radiographs of the included cases were analysed and assessed per follow-up appointment (Table 2). Radiographic images available for assessment included 101 PR and 63 CBCT examinations, of which 57 were initial radiographs, and 107 were follow-up radiographs. In total, approximately 1.9 follow-up radiographs were performed per patient.

**TABLE 2:** Radiologic features of 57 untreated cases of ameloblastoma at initial presentation and follow-up.

Radiologic features	Initial			Follow-up 1			Follow-up 2			Follow-up 3			Follow-up > 4		
	*n*	%	Mean	*n*	%	Mean	*n*	%	Mean	*n*	%	Mean	*n*	%	Mean
**Cases**	57	100	-	57	100	-	24	42	-	11	19	-	6	11	-
**Average duration (months)**	0	-	-	9.16	-	-	27.08	-	-	50.34	-	-	77.24	-	-
**Post-treatment radiographs**	0	-	-	0	-	-	19	33	-	9	16	-	4	7	-
**Borders**															
Well-demarcated	25	43	-	23	40	-	9	37	-	4	36	-	1	17	-
Poorly-demarcated	10	18	-	10	18	-	4	17	-	3	28	-	2	33	-
Focal loss of demarcation	22	39	-	24	42	-	11	46	-	4	36	-	3	50	-
**Locularity**															
Unilocular	15	26	-	11	19	-	4	17	-	1	9	-	0	-	-
Multilocular	41	72	-	44	77	-	19	79	-	10	91	-	6	100	-
Scalloped	1	2	-	2	4	-	1	4	-	0	-	-	0	-	-
**Size**															
Length (mm)	-	-	50.57	-	-	66.19	-	-	68.16	-	-	74.96	-	-	68.47
Height (mm)	-	-	31.58	-	-	43.79	-	-	39.02	-	-	39.40	-	-	63.50
Width (mm)	-	-	53.24	-	-	40.74	-	-	43.07	-	-	45.35	-	-	57.51
Calculated volume (mm^3^)	-	-	85 024	-	-	118 083	-	-	114 549	-	-	133 938	-	-	250 044
**Density**															
Radiolucent	49	86	-	48	84	-	21	88	-	8	73	-	5	83	-
Mixed density	8	14	-	9	16	-	3	12	-	3	27	-	1	17	-
**Tooth effects**															
Root resorption	43	75	-	44	77	-	19	79	-	8	73	-	2	33	-
Tooth impaction	10	18	-	10	18	-	4	17	-	2	18	-	0	-	-
Tooth displacement	35	61	-	35	61	-	15	63	-	7	64	-	3	50	-
Loss of teeth	33	58	-	34	60	-	13	54	-	9	82	-	5	83	-
N/A	3	5	-	3	5	-	1	9	-	0	-	-	0	-	-
**Bone effects**															
Bony expansion	52	91	-	54	95	-	23	96	-	9	82	-	6	100	-
Cortical destruction	46	81	-	52	91	-	22	92	-	9	82	-	6	100	-
Cortical thinning	56	98	-	56	98	-	24	100	-	11	100	-	6	100	-
**Other effects**															
Inferior alveolar nerve	35	61	-	35	61	-	12	50	-	5	46	-	4	-	-
Nasal	2	4	-	2	4	-	1	4	-	1	9	-	1	17	-
Sinus	2	4	-	2	4	-	1	4	-	1	9	-	1	17	-

N/A, not applicable.

Of the 57 patients in the study, none received treatment at the first follow-up, 19 received treatment after two follow-ups, nine after three follow-ups and four after four and/or more follow-ups. Thus, 25 patients had not received treatment at the current institution during the study period.

Radiologically, baseline imaging (initial visit) revealed that most lesions had well-demarcated borders, followed by a focal loss in demarcation. During follow-up examinations, there was a general decrease in the percentage of well-demarcated lesions and an increase in poorly-demarcated lesions or lesions exhibiting a loss of demarcation. Nine cases presented as well-demarcated lesions at baseline imaging with a subsequent change to poorly-demarcated lesions or lesions with a loss in demarcation at follow-up.

**FIGURE 3:**
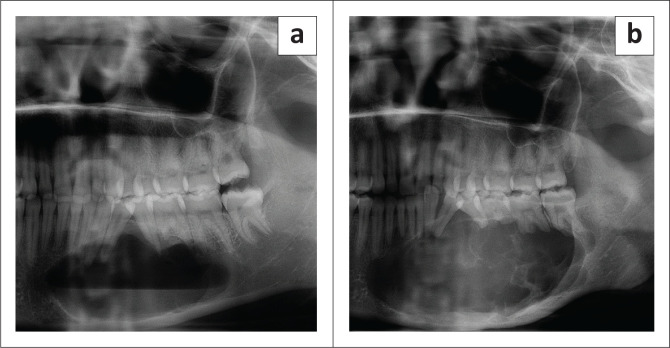
(a) Well-defined unilocular ameloblastoma with scalloped margins causing root resorption and tooth displacement at initial visit. (b) The same lesion after 41 months presenting with a multilocular appearance, an increase in size and more severe root resorption.

Most cases of AB presented with multilocular margins at baseline imaging. The internal density of ABs was radiolucent in the majority of cases. During follow-up visits, the frequency of multilocular lesions increased. Seven cases transformed from an initial unilocular lesion to an eventual multilocular appearance (Figure 3).

**FIGURE 4:**
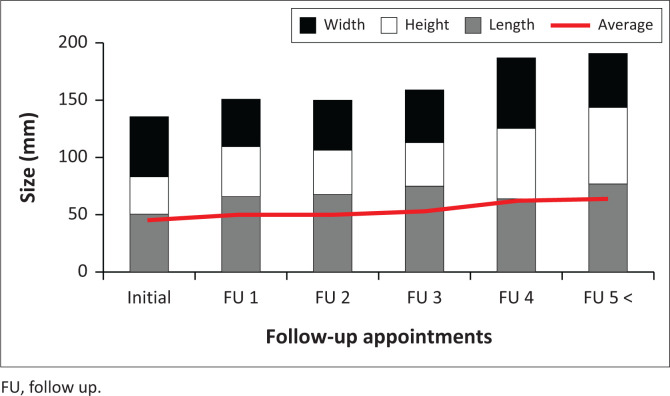
Radiologic size measurement between baseline or initial imaging and subsequent follow-up appointments. The overall dimensions (volume) increased at each follow-up visit.

The average size using the calculated volume was 85 024 mm^3^ at initial presentation and increased to 250 044 mm^3^ at the final follow-up visit. An increase in mean length, height and width from the initial to final visit was seen in most cases (Figure 4). Only five cases showed a slight decrease in size in certain follow-up appointments.

**FIGURE 5:**
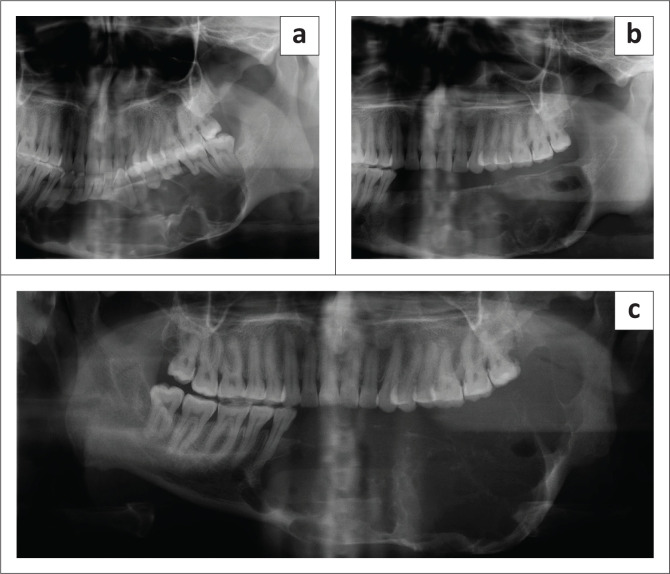
Ameloblastoma at initial presentation (a), 21 months follow-up (b) and 84 months follow-up (c). The lesion showed significant increase in size with loss of associated teeth.

The most common effects on surrounding teeth at baseline imaging were root resorption, tooth displacement and teeth loss. At follow-up visits, the associated loss of teeth in the vicinity of the lesion increased (Figure 5). The most common bone effects at baseline imaging included cortical thinning, bony expansion and cortical destruction. A general trend was noted at follow-up visits, with an increase in all reported bone effects. Additionally, inferior alveolar nerve, maxillary sinus and nasal cavity involvement increased at follow-up visits. Cone-beam computed tomography imaging revealed an additional tooth and bone effect in 10% and 20% of cases, respectively, compared to PR alone.

A regression analysis was performed to determine the change in lesion dimensions over time. Although the physical measurements showed a definite increase in the lesion’s dimensions over time, no significant correlation existed between the change in time and the total radiologic dimensions (*p* = 0.7). There was also no statistically significant relationship for clinical predictors, such as age or gender in the change in ABs’ radiologic features or dimensions. Further regression analysis also showed no statistically significant correlation in length (*p* = 0.158) or height (*p* = 0.393) over time. However, the lower *p*-value found for length shows that length had a stronger correlation to duration compared to ‘overall dimensions’ and ‘height’. This implies ABs with delayed treatment are also more likely to increase in length than height over time.

Regression analysis was also performed to determine the change in measurements between the initial and last visits. The height showed no statistically significant correlation (*p* = 0.1). There was a statistically significant correlation between duration and length (*p* = 0.001). The *r*-square value (0.26) showed that 26% of the variance in the dependent variable could be attributed to the independent variable. Based on these findings, a predictive equation for change in length over time could be established for ABs (change in length over time [mm] = 10.327 + 0.438 × duration [months]). Finally, a statistically significant correlation existed between duration and overall lesion dimensions (volume) when using only the first and last observations per patient (*p* = 0.044).

Both inter- and intra-examiner reliability tests were carried out on the size measurements, with an ICC (*r*) of > 0.8 for all metrics. This shows a strong correlation and a high degree of reliability for both the inter- and intra-examiner reliability tests. To quantify this further, the intra-observer mean difference was also computed as a percentage of the average. This test showed that the average variance was never more than 1%.

## Discussion

Ameloblastoma is the most common benign odontogenic tumour in Africa, with a prevalence of 0.5 per million patients.^2,6,13^ At the current institution, 12 130 head and neck lesions have been diagnosed over the study period, of which 781 were ABs. This translates into a prevalence of 6.4% of all head and neck lesions. Only 57 cases met the inclusion criteria and were included in the final analyses. For this study, ‘delayed treatment’ referred to cases not receiving conservative or radical surgical treatment for the tumour. Most patients received treatment after the second follow-up appointment, after a mean duration of 21 months. At the time of study completion, 32 of the 57 patients had received treatment, two patients who formed part of the study population were still not treated, and 23 were lost to follow-up. The reasons for these cases having delayed treatment were likely multifactorial. Firstly, constraints in healthcare facilities in a developing country mean that malignant cases get preference, with benign lesions only receiving treatment later. Secondly, the other possibility may be patient constraints, including financial problems, transport difficulties, education or language barriers or inadequate knowledge regarding health issues.^14^ However, the current data indicates that this occurs in a minority of cases.

Ameloblastoma usually presents during the third to fifth decades of life with a mean age of 36 years (range: 10–90 years).^3,4,9^ Ameloblastomas is rarely diagnosed in the first two decades, accounting for only 10% – 15% of all reported cases.^3,5,15^ These findings were mirrored in the current study, with 17.5% of cases seen in the first two decades of life. Ameloblastoma shows a slight male predilection with a male-to-female ratio of 1.3:1.^4^ In the current study, a slight female predilection was found. Females presented at a lower median age compared to males. This could be related to the opinion that females present earlier for treatment, as they are more health conscious.^16^ Over 80% of ABs are found in the mandible, with 70% of cases arising in the molar-ramus region, followed by the anterior mandible and posterior maxilla.^3,5^ Involvement of the anterior maxilla is a rare finding.^4,5^ These presentations were mirrored in the current study.

Radiologically, AB has characteristic features, although not entirely pathognomonic.^4^ The most common presentation includes a well-demarcated, multilocular lesion with a honeycomb or soap-bubble appearance.^5,17^ Unilocular presentations are less commonly found. In the mandible, margins are usually well-demarcated, corticated and occasionally scalloped. In contrast, ABs in the maxilla exhibit poorly-demarcated margins, as the lesion tends to grow within, rather than expand the bone. The internal structure of the lesion is radiolucent, with radiopaque bony septa creating internal compartments. Radiologically, there was a general decrease in the percentage of well-demarcated lesions and an increase in cases with loss of demarcation of lesion borders and poorly-demarcated lesions between follow-up examinations. This finding can be explained by ABs’ aggressive nature and persistent growth, where cortical destruction becomes more common over time.^18^ The bony borders lose their demarcation as lesions show increased cortical destruction and soft tissue invasion. The more prolonged the treatment of ABs is delayed, the more tissue infiltration is seen. Of the four cases involving the maxilla, one was well-demarcated at initial presentation but changed to a loss in demarcation at follow-up. The other three cases all showed poorly-demarcated borders or a loss in demarcation.

The current study showed a general decrease in unilocular lesions, with more lesions changing to an eventual multilocular appearance. This finding was also mirrored in other literature. A study by Mariz et al. found that the lesions changed from a unilocular to a multilocular appearance in 10 of the 12 patients they examined (83%).^19^

Ameloblastomas commonly cause root resorption, tooth displacement or impaction.^3^ These findings were mirrored in the current study. The current study found that root resorption, tooth displacement and the number of impacted teeth decreased over time. This could be explained by the increased number of teeth lost in the tumour area. The authors speculate that more teeth were removed near the lesion as time progressed. This could be linked to root resorption resulting in tooth mobility or displacement leading to malocclusion.

Bucco-lingual expansion with cortical perforation is a common finding in most ABs.^3,4,20^ Effects on the dentition include root resorption, tooth displacement or tooth impaction.^3^ Ameloblastomas located in the posterior maxilla can result in encroachment of the maxillary sinuses and, in some instances, intracranial extension.^20^ The current study confirmed this effect with the most common bone effects at baseline imaging, including cortical thinning (98%), bony expansion (91%) and cortical destruction (81%). During follow-up imaging appointments, these effects all increased. Bony margins are significant in treating ABs^6^, with cases with cortical destruction complicating the attainment of tumour-free margins. Cortical destruction also implies soft tissue infiltration, further complicating the treatment and reconstruction.

In the mandible, displacement of the inferior alveolar canal by the tumour is a common finding. Maxillary ABs may cause displacement of the sinus membrane.^4^ This study also confirmed these findings, with increased inferior alveolar canal displacement (61% to 67%) and maxillary sinus or nasal cavity (4% to 17%) involvement in follow-up imaging visits. This implies that as time progresses, the involvement of critical anatomical structures increases, further complicating management.

Ideally, ABs should be treated at the initial presentation, but this is not always the reality in developing countries with financial or economic constraints. Only isolated studies offer any information about the growth of ABs. Ameloblastoma exhibits an aggressive growth pattern^18^ that is initially slow but accelerates later.^21^ Because of this, it is challenging to determine the growth characteristics of AB. Factors associated with a more rapid growth rate and poorer prognosis include maxillary ABs, conventional ABs, mural unicystic AB subtypes, older patients and suboptimal treatment.^21^ The current study found no clinical predictors, such as age or gender, for the change in the radiologic features or dimensions of ABs. One study found that ABs have an average annual growth rate of 40.4%. This was a much lower growth rate than other studies (88% per year) because these studies only predicted the growth rate by relying on patient information about when the lesions started.^21^ The average size using the calculated volume increased from the initial presentation to the final available follow-up radiograph. At each follow-up visit, there was a significant increase in the overall dimensions of the lesion. There was, however, no significant linear correlation between the change in dimensions or mass between individual follow-up visits. This implies that one cannot predict how an AB will grow or change between follow-up appointments and that ABs do not grow linearly. Ameloblastoma has been described as a benign tumour with intermittent growth.^20^ However, when the lesion dimensions were analysed from the first to the last visit, a statistically significant relationship was found over duration or time. The growth in length had the strongest significant correlation over time with a prediction of change in length over the duration. The overall dimensions or volume of lesions also showed statistically significant and predictive growth over time. There were exceptions in five cases that slightly decreased in size between certain follow-up appointments. This could be attributed to central necrosis in large tumours or compression of cystic spaces after extractions.

Limitations of the current study included the different imaging modalities used to compare the lesion size (PRs and CBCT images). In addition, because of the study’s retrospective nature, not all patients could be included in the study owing to lost information or inadequate radiographic examinations. Limitations in the evaluation process were lesions with a loss in demarcation, which made accurate measurements difficult. An additional shortcoming was the follow-up periods of patients not being standardised. Not all patients came for follow-up after the same period, meaning that the growth rate of these lesions was challenging to assess. Lesion size estimation on PRs is problematic because of unequal magnification in cases with positioning errors.^22^ Using the teeth as references to assess variable magnifications between the left and right side in cases of AB should be considered unreliable, as the tumour may displace teeth in buccal or lingual dimensions depending on the growth pattern. Stramotas et al.^23^ showed that linear measurements on PR are affected more when the occlusal plane is titled in a superior-inferior dimension. Nonetheless, considering this limitation, the margin of error was still minimal, within 1 mm. This finding was also mirrored in a study by Nikneshan et al.^24^ With the advent and routine utilisation of more modern imaging modalities, the usefulness of PR should not be disregarded as they help estimate invasion and root resorption at a relatively low radiation dose. In addition, AB size estimation has previously been assessed on PR to indicate AB growth.^19,21^

Two-dimensional limitations of radiographs, such as distortion and superimposition, are negated using a superior three-dimensional imaging projection. Cone-beam computed tomography scans are mandatory for three-dimensional lesion demarcation, including accurate assessment of relationships with skull structures.^25,26^ Other tooth and bone effects were identified in 10% and 20% of cases, respectively, because of CBCT imaging. This highlights the importance of CBCT as an imaging modality in cases of AB. The lesion dimensions of CBCT images may also be an inaccurate reflection if the head tilt is altered or not standardised between scans. Therefore, the results of all imaging modalities should be interpreted with a background understanding of the physics and the limitations of the imaging modalities used. Irrespective of these limitations, the other findings of the current study related to the changes in borders, cortication and locularity are insightful.

## Conclusion

This study is the first to report on the radiological progression of ABs with delayed treatment within a large sample size. Overall, the lesions increased in size with increased effects on the teeth, bone and surrounding structures. This may complicate the management of these patients as more extensive surgical and reconstructive procedures are necessary. These findings may assist clinicians in emphasising the need for early diagnosis and management of these patients because of the growth potential of these tumours.
